# Pharmacological treatment of MASH

**DOI:** 10.1007/s00125-026-06796-1

**Published:** 2026-07-17

**Authors:** Norbert Stefan, Giovanni Targher

**Affiliations:** 1 Internal Medicine IV, Department of Endocrinology, Diabetology and Nephrology, University Hospital Tübingen, Tübingen, Germany; 2 Institute of Diabetes Research and Metabolic Diseases (IDM) of Helmholtz Munich at the University of Tübingen, Tübingen, Germany; 3 German Center for Diabetes Research (DZD), Tübingen, Germany; 4 Department of Medicine, University of Verona, Verona, Italy; 5 Metabolic Diseases Research Unit, IRCCS Sacro Cuore – Don Calabria Hospital, Negrar di Valpolicella (VR), Italy

**Keywords:** Cardiometabolic diseases, MASLD, Metabolic dysfunction-associated steatohepatitis, Metabolic dysfunction-associated steatotic liver disease, Pharmacological treatment, Review, Type 2 diabetes

## Abstract

Metabolic dysfunction-associated steatotic liver disease (MASLD) includes a spectrum of progressive liver conditions ranging from isolated steatosis to metabolic dysfunction-associated steatohepatitis (MASH), advanced fibrosis and cirrhosis. Currently, MASLD is the leading cause of chronic liver disease worldwide. MASLD is strongly associated with type 2 diabetes, cardiovascular disease, chronic kidney disease and certain extrahepatic cancers. MASLD shares a common pathogenesis with cardiometabolic diseases, especially type 2 diabetes, primarily driven by unhealthy dietary habits, dysfunctional adipose tissue, insulin resistance and low-grade inflammation. Substantial heterogeneity in the pathophysiology of MASLD may influence its rate of progression, its relationship with cardiometabolic diseases and its treatment response. In addition to lifestyle interventions, including a healthy low-energy diet and increased physical activity levels, pharmacological treatment of MASLD/MASH is recommended. For individuals with type 2 diabetes and MASLD/MASH, treatment should preferably include glucagon-like peptide-1 (GLP-1) receptor agonist-based therapies and sodium–glucose cotransporter 2 (SGLT2) inhibitors, which have been shown to improve MASLD/MASH and provide established cardiorenal benefits. In this narrative review, we assess the efficacy of these pharmacotherapies and discuss other treatment approaches for MASLD/MASH, with a focus on their metabolic benefits.

**Graphical Abstract:**

**Figure:**
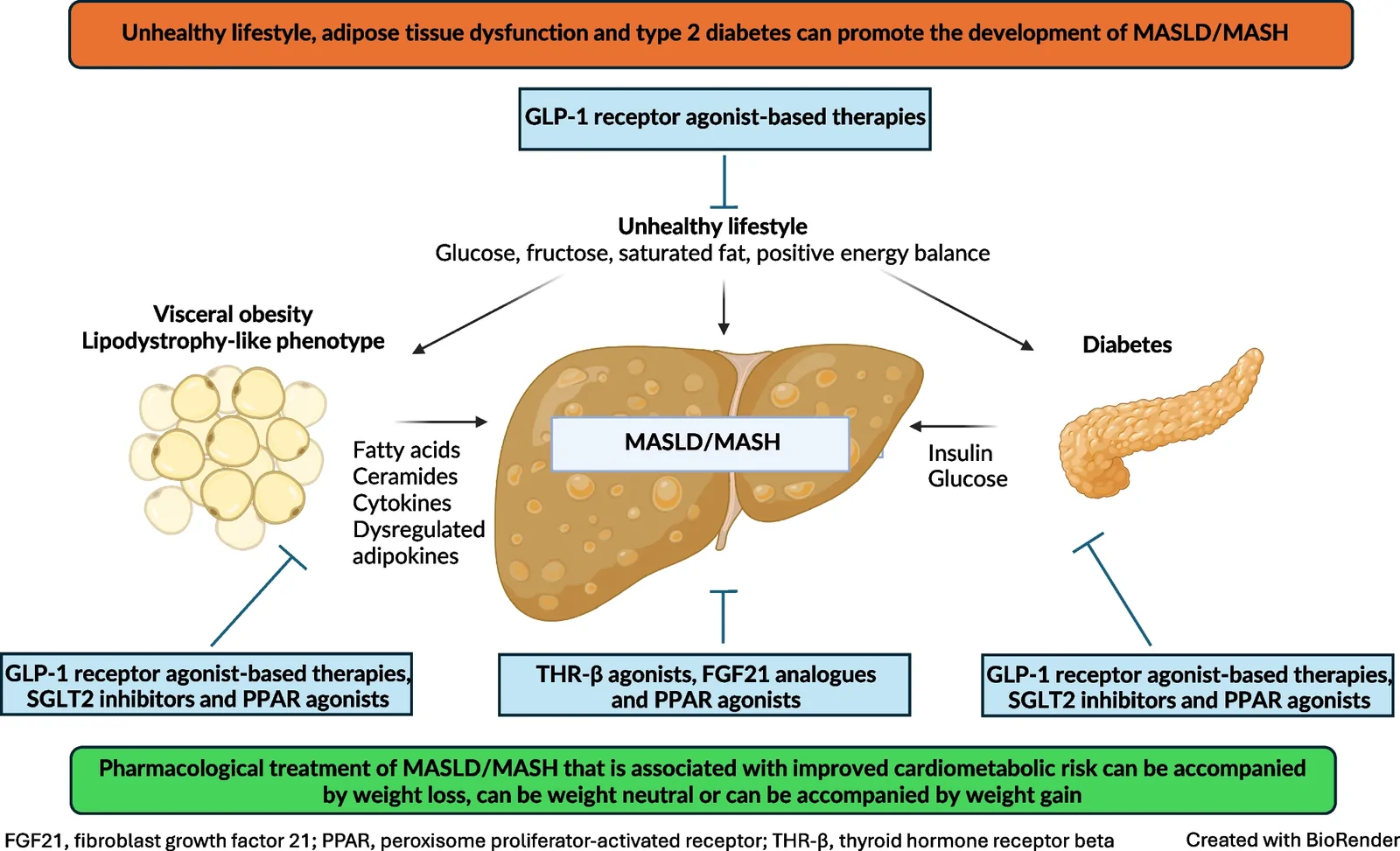

**Supplementary Information:**

The online version contains a slideset of the figures for download available at https://doi.org/10.1007/s00125-026-06796-1.

## Introduction

Metabolic dysfunction-associated steatotic liver disease (MASLD), formerly termed non-alcoholic fatty liver disease (NAFLD), has rapidly become the most common chronic liver disease, affecting up to 35–40% of the global adult population [1]. MASLD includes a spectrum of liver conditions, progressing from isolated steatosis to metabolic dysfunction-associated steatohepatitis (MASH), advanced fibrosis and cirrhosis, which may progress to hepatocellular carcinoma [1]. The global prevalence of MASLD and its more progressive form, MASH, is even higher among people with type 2 diabetes. In a meta-analysis of 156 observational studies that included ~1.8 million individuals with type 2 diabetes, the global prevalence rates of MASLD and MASH were 65% (95% CI 62%, 68%) and 31.5% (95% CI 17%, 51%), respectively [2].

MASLD is an increasingly important contributor to liver-related and extrahepatic morbidity and mortality worldwide [3, 4]. Cardiovascular disease is the leading cause of mortality among individuals with MASLD, followed by extrahepatic cancers (primarily gastrointestinal, breast and gynaecological cancers) and liver-related complications, including cirrhosis, hepatic decompensation and hepatocellular carcinoma [3, 4].

First-line treatment of MASLD/MASH includes lifestyle modifications, such as weight-reducing diets, physical exercise and avoidance of alcohol, as well as management of type 2 diabetes, obesity, hypertension and dyslipidaemia [5]. Over the past decade, many MASH therapeutic trials have been unsuccessful because they failed to achieve clinically meaningful improvements in histological liver inflammation and fibrosis. These failures may reflect the complex, multifactorial pathophysiology of this liver disease, challenges in trial design, and limitations in the available drug mechanisms. Notably, in March 2024 and August 2025, respectively, resmetirom (an oral, liver-directed thyroid hormone receptor-β (THR-β)-selective agonist) and subcutaneous semaglutide 2.4 mg/week were granted accelerated approval by the US Food and Drug Administration (FDA) for the treatment of adults with non-cirrhotic MASH and moderate-to-advanced fibrosis in conjunction with lifestyle modification [4]. Resmetirom and semaglutide were subsequently approved by the European Medicines Agency (EMA) in August 2025 and March 2026, respectively [4].

In this narrative review, we discuss pharmacological therapeutic options for MASH with a focus on newly approved or promising metabolism-based pharmacotherapies currently being evaluated in late-phase RCTs for the treatment of this common and burdensome liver disease. These therapies may beneficially affect MASLD/MASH and its liver-related and extrahepatic (cardiometabolic) clinical outcomes. Clinical pharmaceutical trials in MASLD/MASH were identified on 31 December 2025 using PubMed and ClinicalTrials.gov using the following search terms: ‘metabolic dysfunction-associated steatotic liver disease’ OR ‘MASLD’ OR ‘metabolic dysfunction-associated steatohepatitis’ OR ‘MASH’ OR ‘non-alcoholic fatty liver disease’ OR ‘NAFLD’ OR ‘non-alcoholic steatohepatitis’ OR ‘NASH’ AND ‘liver histology’ OR ‘liver biopsy’; the following filters were applied: Clinical Trial, Phase II, Clinical Trial, Phase III, Randomised Controlled Trial. Free full-text terms were ‘humans’ and ‘adult 19+ years’. We considered only English-language articles published in peer-reviewed journals.

## Clinical endpoints in MASH trials

To determine the efficacy of a drug for the treatment of MASH and liver fibrosis, both the FDA and the EMA have recommended using a long-term composite endpoint that includes all-cause mortality, histological diagnosis of cirrhosis, hepatic decompensation events, and model for end-stage liver disease score assessments. However, because of the time required to assess long-term clinical endpoints, both regulatory agencies have advised earlier evaluation of intermediate/surrogate histological endpoints to accelerate regulatory approval, given the unmet medical need for MASH pharmacotherapies [6].

Liver histological endpoints—specifically, resolution of MASH without worsening of fibrosis, and improvement in liver fibrosis by one or more stage without worsening of MASH—are considered appropriate intermediate/surrogate endpoints in regulatory and reimbursement settings, based on current regulatory guidelines. Both regulatory agencies accept these two histological endpoints for accelerated approval of new candidate drugs for the treatment of MASH and liver fibrosis [6].

However, the growing global burden of MASLD and MASH, along with the urgent need for effective pharmacotherapies, has driven the adoption of non-histological surrogate endpoints for treatment assessment in MASH [7]. Notably, on 27 August 2025, the FDA accepted a letter of intent for the qualification of liver stiffness measurement by vibration-controlled transient elastography (VCTE) as a reasonably likely surrogate endpoint for RCTs in adults with MASH and moderate-to-advanced fibrosis [8]. VCTE is a reliable non-invasive test for assessing liver fibrosis that has been shown to accurately predict all-cause mortality and long-term liver-related clinical outcomes in individuals with MASLD [9–11]; VCTE can also provide a safer, more accessible and pragmatic approach for monitoring liver disease progression and treatment response [7, 12]. The 2024 EASL/EASD/EASO guidelines strongly support a two-step risk stratification approach using non-invasive tests for staging advanced liver fibrosis in MASLD: blood-based scores (such as the Fibrosis-4 [FIB-4] index) act as first-line screening, while VCTE is used as a second-line or confirmatory test to rule out/in advanced liver fibrosis [5]. Recent literature provides a comprehensive discussion of the main challenges involved in applying these non-invasive tests, as well as potential future research avenues for monitoring liver disease progression and treatment response in MASLD [7, 12].

Overall, the recent FDA acceptance of the letter of intent on VCTE marks a paradigm shift towards the use of non-invasive tests, rather than invasive methods such as liver biopsy, for routine monitoring of liver disease, with important implications for trial design, reimbursement and clinical guidelines. Evaluating new pharmacotherapies for MASH without repeated liver biopsies may improve trial recruitment, help overcome difficulties in conducting adequately powered clinical trials and accelerate drug development for individuals living with MASH [13]. Furthermore, given the systemic nature of this metabolic liver disease, in addition to non-histological surrogate endpoints, regulatory agencies should also consider cardiometabolic endpoints for conditional approval of new drug candidates for the treatment of MASH and liver fibrosis [13].

## Pathogenesis of MASLD

The pathogenesis of MASLD is complex and not fully understood. According to the ‘multiple parallel hits hypothesis’, MASLD is not caused by a single factor but by multiple simultaneous insults (‘hits’), in which adipose tissue inflammation, metabolic factors (mainly insulin resistance), hepatic lipotoxicity, alterations in gut microbial function, and genetic predisposition play central roles [14]. The hallmark of MASLD is the accumulation of hepatic lipid droplets that act as organelles, storing inert and toxic lipids and triggering activation of stress pathways, inflammation and cell death, thereby leading to liver damage and fibrosis [3]. This accumulation of intracellular lipid droplets results from an imbalance between lipid uptake, synthesis (hepatic de novo lipogenesis) and breakdown (hepatic β-oxidation). It is beyond the scope of this review to provide a detailed overview of the key pathophysiological mechanisms underlying MASLD; a comprehensive discussion is available in the recent literature [3, 15, 16].

**Fig. 1:**
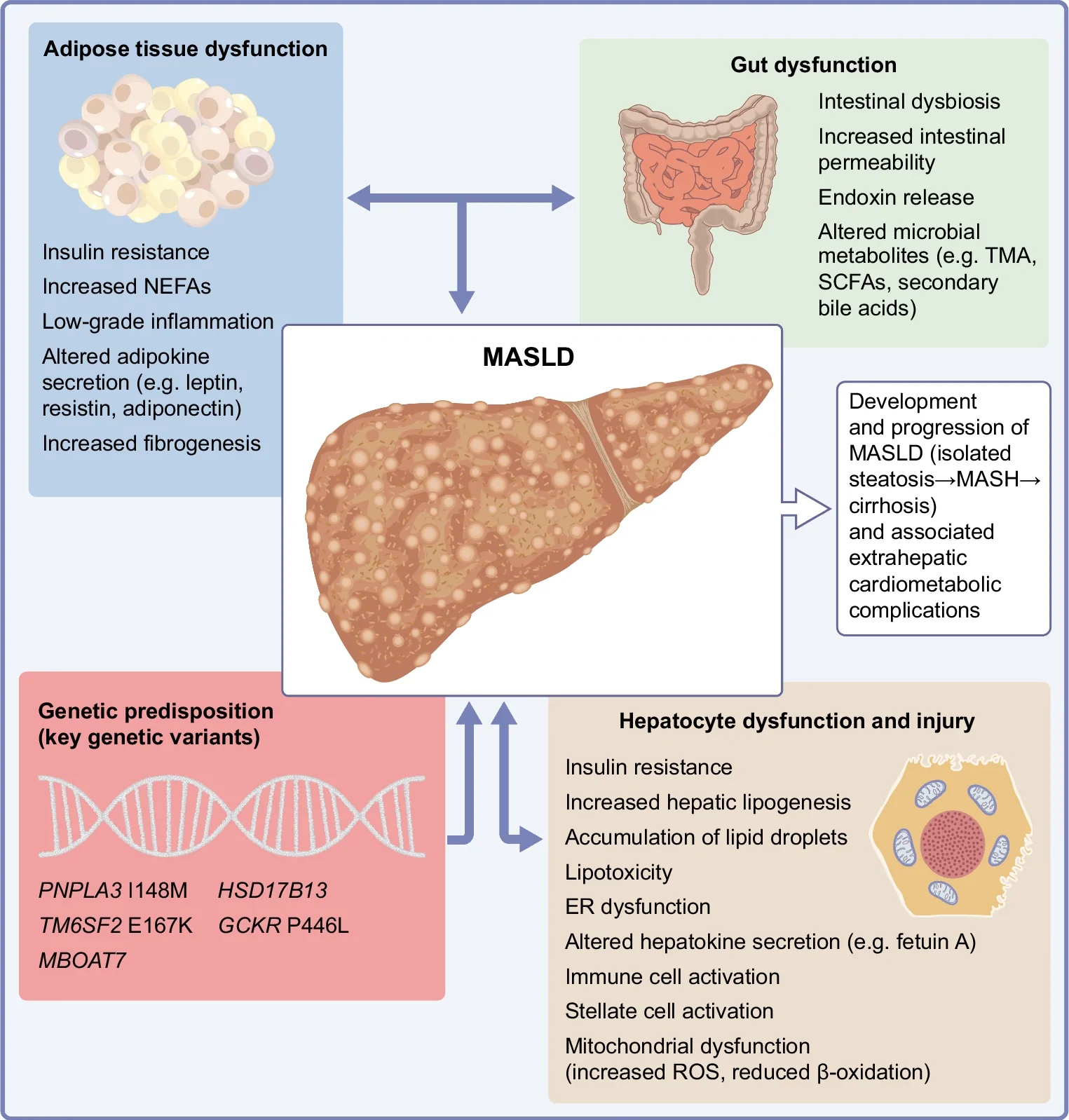
Overview of the key factors in the pathophysiology of MASLD. Dysfunctional adipose tissue, characterised by more severe insulin resistance, low-grade inflammation, fibrogenesis and an altered adipokine production profile, is strongly associated with MASLD. Collectively, these alterations can result in an increased flux of NEFAs into the liver, promoting hepatic steatosis and exacerbating systemic chronic inflammation and cardiometabolic dysfunction. Simultaneously, intestinal dysfunction, driven by gut dysbiosis and loss of intestinal barrier integrity, results in increased endotoxin release and altered production of other microbial metabolites, all of which have been shown to promote the development and progression of MASLD. Common genetic risk variants, such as *PNPLA3* I148M, may increase the risk of developing MASLD and its extrahepatic complications. In hepatocytes, increased hepatic de novo lipogenesis, excess NEFA uptake and insulin resistance drive lipid droplet accumulation. Once established, intracellular lipid droplets are thought to displace the cell’s nucleus, induce endoplasmic reticulum stress and be closely associated with the generation of lipotoxic lipid intermediates, such as ceramides and diacylglycerol. Simultaneously, mitochondrial dysfunction promotes oxidative stress and reduces fatty acid oxidation, further driving hepatic steatosis and inflammation. Collectively, these processes induce hepatocyte dysfunction and activate resident immune cells and stellate cells, driving progression from isolated steatosis to MASH, advanced fibrosis and cirrhosis. ER, endoplasmic reticulum; ROS, reactive oxygen species; SCFAs, short-chain fatty acids; TMA, trimethylamine. This figure is available as part of a downloadable slideset

Figure 1 provides a framework for understanding how perturbations in adipose tissue function, lipid metabolism, proinflammatory signalling, gut–liver axis crosstalk and genetic predisposition (e.g. variants in *PNPLA3* [I148M] and *TM6SF2* [E167K]) can interact to promote MASLD, systemic cardiometabolic dysfunction and ultimately progression to liver fibrosis and advanced chronic disease states. Behavioural factors, such as a sedentary lifestyle, high fructose intake (primarily from sugar-sweetened beverages) and high-energy diets, especially those high in saturated fats, sugars and processed foods, may also contribute to the development of MASLD [4].

## Results of Phase II(b), III and IV clinical trials in individuals with biopsy-confirmed MASH and moderate-to-advanced fibrosis

The primary aim of MASLD treatment is to induce MASH resolution without progression of liver fibrosis and to improve hepatic fibrosis without worsening of MASH. Effective management of type 2 diabetes and other metabolic comorbidities (obesity, dyslipidaemia and hypertension) is also important to reduce the risk of MASLD progression and the development of extrahepatic cardiometabolic and oncological complications [3–5]. Histological improvements in MASH and liver fibrosis may ultimately reduce the risk of long-term liver-related events (such as new-onset cirrhosis, hepatic decompensation, hepatocellular carcinoma and liver-related mortality) [5, 17]. A lifestyle intervention aiming for weight loss of >5% is considered effective for decreasing hepatic steatosis, while weight loss of 7–10% and >10% is thought to improve MASH and liver fibrosis, respectively [18]. Such weight loss is often achieved through a low-energy diet and an increase in physical activity levels (preferably at least 150 min of moderate-intensity aerobic exercise or 75–150 min of vigorous-intensity exercise weekly) [3–5]. In addition, a Mediterranean diet characterised by high intake of vegetables, whole grains, fruit, fish and olive oil, and reduced intake of ultra-processed foods, saturated fats and refined sugars, improves liver histology in MASLD/MASH [19]. Effective lifestyle interventions are also expected to significantly reduce the increased cardiometabolic risk observed in MASLD/MASH [4, 5].

**Fig. 2:**
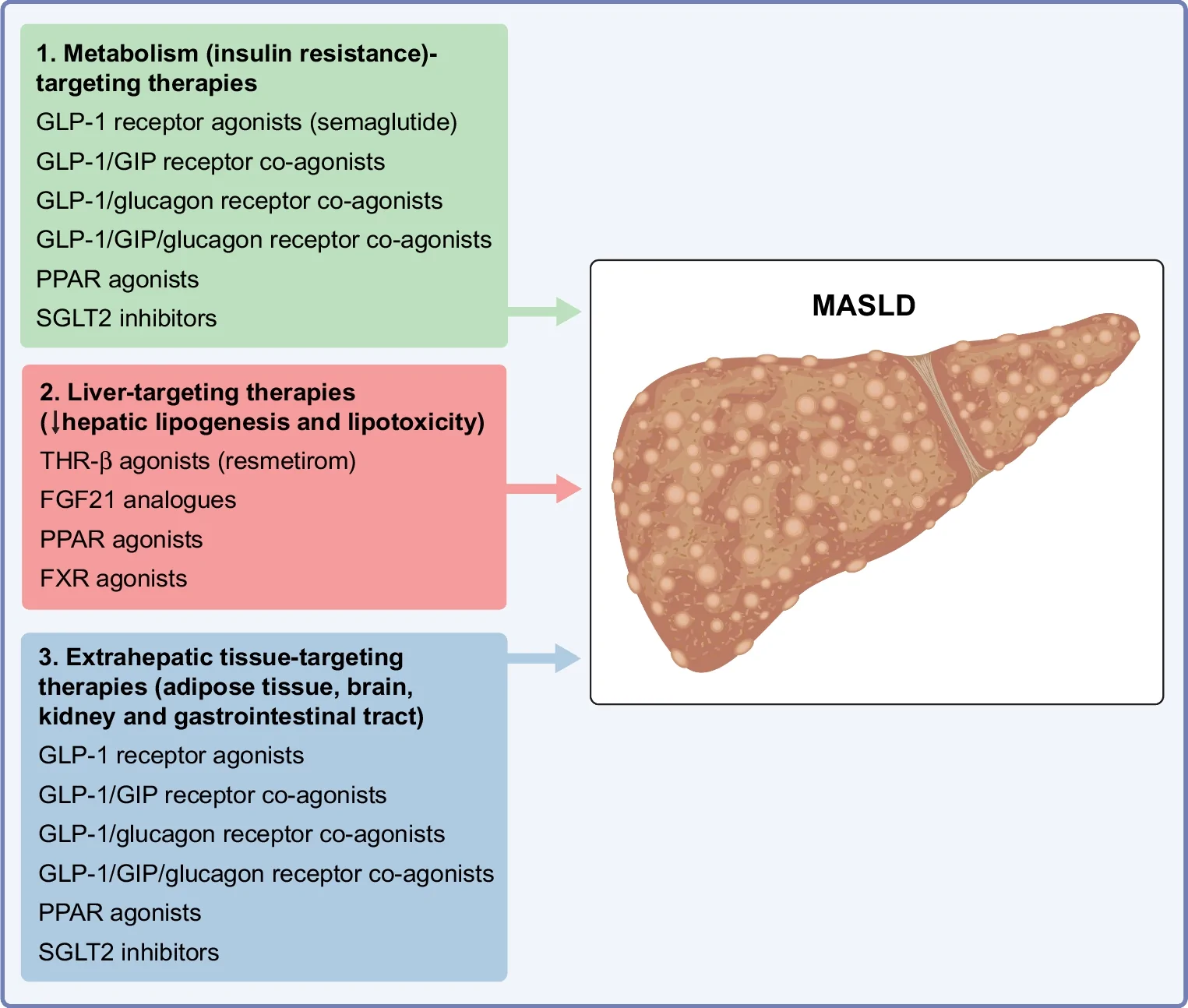
Summary of selected mechanisms of action targeted by MASH pharmacotherapies. GLP-1 receptor agonist-based therapies, SGLT2 inhibitors and PPAR agonists effectively treat MASH by reducing hyperglycaemia and improving insulin sensitivity. THR-β agonists (resmetirom), FGF21 analogues, PPAR agonists and FXR agonists (not discussed in this review) improve liver pathology in individuals with MASH by decreasing hepatic lipogenesis and lipotoxicity. GLP-1 receptor agonist-based therapies, SGLT2 inhibitors and PPAR agonists are also effective for the treatment of MASH through their beneficial effects on adipose tissue, brain, kidney and the gastrointestinal tract. This figure is available as part of a downloadable slideset

However, for most people living with MASLD/MASH, maintaining a healthy lifestyle over the long term is difficult and weight regain is often observed. Therefore, pharmacological treatment of MASLD/MASH is considered an important additional cornerstone in its management. Liver-directed pharmacotherapy specifically targeting hepatic inflammation and fibrosis has been previously discussed [20, 21]; here, we focus on metabolism-based treatment approaches in MASH (Fig. 2), assuming that they also beneficially affect cardiometabolic comorbidities, which are often increased in individuals with type 2 diabetes and MASLD/MASH because of the shared underlying metabolic abnormalities [3, 22]. This shared pathophysiology has led to several medical guidelines on the management of MASLD/MASH strongly recommending the use of glucagon-like peptide-1 (GLP-1) receptor agonist-based therapies, alone or in combination with sodium–glucose cotransporter 2 (SGLT2) inhibitors, as first-line therapeutic options for individuals with type 2 diabetes and MASLD [5, 17]. In their Standards of Care published in December 2025, the ADA recommends a GLP-1 receptor agonist with demonstrated benefits in MASH or a dual glucose-dependent insulinotropic polypeptide (GIP) and GLP-1 receptor agonist with potential benefits in MASH for glycaemic management in people with type 2 diabetes, MASLD and overweight or obesity [23]. In addition, a recent consensus report by the ADA on the screening and management of MASLD recommends that pioglitazone, GLP-1 receptor agonists, dual GIP and GLP-1 receptor agonists or SGLT2 inhibitors be used to treat people living with type 2 diabetes and MASLD [24]. In people with type 2 diabetes and MASH, pioglitazone, a GLP-1 receptor agonist or a dual GIP and GLP-1 receptor agonist should be used for therapy, and resmetirom should be considered by a hepatologist with expertise in MASH and within the context of an interprofessional team approach [24].

In the remainder of this section, we specifically discuss the results of late-phase RCTs on the treatment of adults with biopsy-confirmed non-cirrhotic MASH and liver fibrosis, which may improve liver-related and extrahepatic (mostly cardiometabolic) clinical outcomes. The results of Phase IIa clinical trials without repeated liver biopsies are not discussed in this review (e.g. a 48-week, placebo-controlled Phase IIa trial of the triple hormone receptor agonist retatrutide in individuals with MRI-defined MASLD [25]). Because most individuals with MASLD are living with overweight or obesity, weight loss is a primary treatment target in this population. However, an estimated 7–20% of those with MASLD have a normal body weight (so-called ‘lean MASLD’) [26]. Most individuals with lean MASLD are metabolically unhealthy and have insulin resistance, characterised by a lipodystrophy-like phenotype, primarily driven by low gluteofemoral and leg fat mass [27], which is an independent determinant of cardiometabolic risk [28]. Therefore, we highlight pharmacological approaches for MASLD that are weight-neutral or associated with weight reduction or weight gain (mostly an increase in subcutaneous adipose tissue) (see Text box, Summary of pharmacological treatment of MASH in people with type 2 diabetes). Furthermore, because in most published clinical trials on the treatment of MASLD or MASH, >50% of participants had type 2 diabetes, and because this review focuses on metabolism-based therapeutic approaches in MASH, we also report the treatment effects on HbA_1c_ levels and insulin resistance (Table 1).

**Figure:**
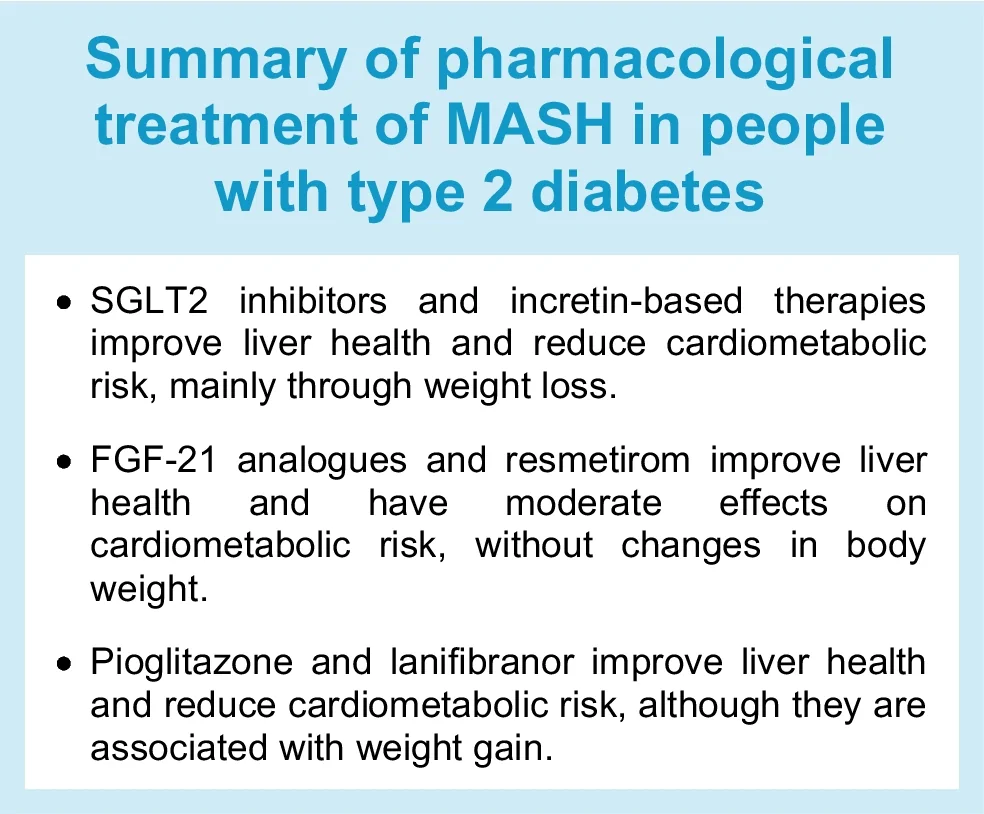

**Table 1:** Selected agents with beneficial metabolic effects that have been studied in active RCTs in adults with biopsy-proven non-cirrhotic MASH and fibrosis

Mode of action and drug name	Population	Trial phase	Treatment duration (weeks)	Type 2 diabetes (%)	MASH resolution	Hepatic and metabolic effects of active treatment vs comparator^a^						
						Steatosis score	Inflamma-tion score	Fibrosis score	Liver enzymes	Body weight	HbA_1c_	Insulin resistance
SGLT2 inhibitors												
Tofogliflozin 20 mg/day vs glimepiride 0.5 mg [31]	Biopsy-proven MASLD	Phase IV	48	100	Yes	–	–	–	↓	↓	↓	NA
Dapagliflozin 10 mg/day vs placebo [32]	Biopsy-proven MASH	Phase III	48	45	Yes	↓	↓	↓	─ ALT, AST; ↓ GGT	↓	↓	↓
GLP-1 receptor agonists												
Semaglutide 0.1 mg, 0.2 mg or 0.4 mg/day vs placebo [35]	Biopsy-proven MASH + liver fibrosis (F1–F3)	Phase IIb	72	62	Yes	↓	↓	─	↓	↓	↓	NA
Semaglutide 2.4 mg/week vs placebo [36]	Biopsy-proven MASH + liver fibrosis (F2 or F3)	Phase III	72	56	Yes	NA	NA	↓	↓	↓	↓	↓
GIP/GLP-1 receptor co-agonist												
Tirzepatide 5 mg, 10 mg or 15 mg/week vs placebo [43]	Biopsy-proven MASH + liver fibrosis (F2 or F3)	Phase IIb	52	58	Yes	↓	↓	↓	↓	↓	↓	NA
Glucagon/GLP-1 receptor co-agonist												
Survodutide 2.4, 4.8 or 6.0 mg/week vs placebo [45]	Biopsy-proven MASH + liver fibrosis (F1–F3)	Phase IIb	24	39	Yes	↓	↓	–	↓	↓	↓	NA
FGF21 analogues												
Pegozafermin 15 mg or 30 mg weekly or 44 mg once every 2 weeks vs placebo [48]	Biopsy-proven MASH + liver fibrosis (F2 or F3)	Phase IIb	24	66	Yes	↓	↓	↓	↓	–	–	NA
Efruxifermin 28 mg or 50 mg/week vs placebo [49]	Biopsy-proven MASH + liver fibrosis (F2 or F3)	Phase IIb	96	70	Yes	↓	↓	↓	↓	–	–	↓
THR-β receptor agonist												
Resmetirom 80 mg or 100 mg/day vs placebo [51]	Biopsy-proven MASH + liver fibrosis (F1B, F2 or F3)	Phase III	52	67	Yes	↓	↓	↓	↓	–	–	–
PPAR agonists												
Pioglitazone (PPARγ) 30 mg or 45 mg/day vs placebo [56]	Biopsy-proven MASH and liver fibrosis	Meta-analysis of Phase II trials	24–96	16	Yes	↓	↓	↓	↓	↑	↓	↓
Lanifibranor (PPARα/δ/γ; pan-PPAR agonist) 800 mg or 1200 mg/day vs placebo [58]	Biopsy-proven MASH (76% moderate or advanced fibrosis)	Phase IIb	24	42	Yes	↓	↓	↓	↓	↑	↓	↓

Some of the information provided in this table has been previously reported in Stefan et al [22]

^a^↓ or ↑, statistical difference vs comparator; –, not altered

ALT, alanine aminotransferase; F1, mild fibrosis; F1B, moderate perisinusoidal fibrosis; F2, moderate fibrosis; F3, severe fibrosis; GGT, γ-glutamyl transferase; GIP, glucose-dependent insulinotropic polypeptide; GLP, glucagon-like peptide; NA, not assessed; PPAR, peroxisome proliferator-activated receptor; SGLT2, sodium–glucose cotransporter 2; THR-β, thyroid hormone receptor-beta

### SGLT2 inhibitors

SGLT2 inhibitors, originally introduced to reduce chronic hyperglycaemia in individuals with type 2 diabetes, consistently reduce the risk of heart failure events and cardiovascular death in those with type 2 diabetes, heart failure, chronic kidney disease or cardiovascular disease [29]. By inducing glycosuria, SGLT2 inhibitors lower blood glucose and insulin levels, thereby improving insulin sensitivity, reducing hepatic de novo lipogenesis and promoting moderate weight loss. Furthermore, SGLT2 inhibitors may also promote weight loss by activating the liver–brain–adipose neurocircuitry. Finally, SGLT2 inhibitors modulate the composition of the gut microbiota. These agents increase the abundance of beneficial short-chain fatty acid-producing bacteria and reduce harmful metabolite production by the gut microbiota [30]. Therefore, SGLT2 inhibitors are a promising therapeutic option for managing MASLD/MASH, particularly in individuals with type 2 diabetes. As such, in a randomised, open-label, active-controlled Phase IV trial conducted in adults with biopsy-proven MASLD and type 2 diabetes, tofogliflozin induced greater improvements in steatosis, hepatocellular ballooning, lobular inflammation and fibrosis than glimepiride, although these differences were not statistically significant (all *p*=0.06–0.17) [31]. Furthermore, in a small Phase III randomised, placebo-controlled trial (the DEAN trial) involving 154 adults with biopsy-confirmed MASH with or without type 2 diabetes, dapagliflozin 10 mg daily for 48 weeks was more effective than placebo in terms of MASH resolution (23% vs 8%, respectively) and fibrosis improvement (45% vs 20%, respectively) [32]. In both trials, SGLT2 inhibitors reduced body weight and HbA_1c_ levels more effectively than the control.

In a recent meta-analysis of eight active-comparator, new-user cohort studies with aggregate data on 626,104 individuals with type 2 diabetes (397,806 new users of SGLT2 inhibitors and 228,298 new users of other glucose-lowering agents), the new use of SGLT2 inhibitors was associated with a significantly lower risk of liver-related events (defined as a composite of new-onset hepatocellular carcinoma, hepatic decompensation, liver transplantation or liver-related deaths) and liver-related deaths. Notably, the significant risk reduction in liver-related events was observed when comparing SGLT2 inhibitors with dipeptidyl peptidase 4 inhibitors, metformin or pioglitazone, but not with GLP-1 receptor agonists [33].

### GLP-1 receptor agonists

GLP-1 receptor agonists were originally introduced to treat type 2 diabetes. Treatment with GLP-1 receptor agonists has been associated with a significant reduction in major adverse cardiovascular events and mortality, and they are considered to improve heart failure and protect against progressive chronic kidney disease. In addition, these agents are approved for the treatment of obesity. In addition to moderately increasing insulin secretion and substantially reducing body weight, GLP-1 receptor agonists reduce systemic low-grade inflammation primarily through weight loss, neuronal GLP-1 receptor activation and GLP-1 receptor activation on T cells [34]. In a Phase IIb trial of 320 adults with obesity and biopsy-confirmed MASH and fibrosis stages F1–F3, subcutaneous semaglutide (0.1 mg, 0.2 mg or 0.4 mg daily for 72 weeks) resulted in a significantly higher proportion of participants with MASH resolution without worsening of fibrosis than placebo (40%, 36% and 59% in participants treated with 0.1 mg, 0.2 mg or 0.4 mg daily, respectively, compared with 17% in the placebo group). Conversely, semaglutide failed to improve liver fibrosis [35]. Recently, in part 1 of the Phase III, randomised, double-blind, placebo-controlled ESSENCE trial involving 800 adults with obesity and biopsy-proven MASH and fibrosis stages 2 or 3, subcutaneous semaglutide at a dose of 2.4 mg weekly for 72 weeks was superior to placebo with respect to histological resolution of MASH without worsening of fibrosis (62.9% vs 34.3%, respectively) and improvement in liver fibrosis with no worsening of MASH (36.8% vs 22.4%, respectively) [36]. In both trials, semaglutide significantly reduced body weight and improved plasma lipid profile and HbA_1c_ levels, and in the ESSENCE trial, insulin resistance (as estimated by HOMA-IR) was also improved compared with placebo. In a small Phase IIb placebo-controlled trial involving 71 participants with obesity and MASH-related compensated cirrhosis, semaglutide 2.4 mg once weekly for 48 weeks did not significantly improve liver fibrosis or achieve MASH resolution compared with placebo [37]. Based on the results of part 1 of the Phase III ESSENCE trial, the FDA on 15 August 2025 [38] and the EMA on 26 March 2026 [39] conditionally approved semaglutide 2.4 mg/week for the treatment of adults with non-cirrhotic MASH and moderate-to-advanced fibrosis. Evaluation of longer-term data from part 2 of the ESSENCE trial (NCT04822181) is ongoing to assess the effects of semaglutide 2.4 mg/week on liver-related events over 240 weeks.

### GLP-1/GIP receptor co-agonists

GLP-1/GIP receptor co-agonists have additive beneficial metabolic effects [40]. Tirzepatide is the first GLP-1/GIP receptor co-agonist approved for the treatment of type 2 diabetes and obesity. Tirzepatide is superior to GLP-1 receptor agonists in lowering HbA_1c_ levels in individuals with type 2 diabetes and in reducing body weight in those with and without type 2 diabetes [41, 42]. Metabolic benefits of tirzepatide, independent of weight loss, include improved insulin sensitivity, increased insulin secretion and increased glucagon-induced lipid oxidation. Furthermore, during hyperinsulinaemia, GIP receptor signalling in adipose tissue enhances lipid storage in white adipose tissue, thereby reducing ectopic lipid deposition in the liver and skeletal muscles [40]. In a Phase IIb, dose-finding, randomised, placebo-controlled trial (SYNERGY-NASH) involving 190 participants with obesity and biopsy-confirmed MASH and stage F2 or F3 fibrosis, once-weekly subcutaneous tirzepatide (5 mg, 10 mg or 15 mg for 52 weeks) resulted in significantly higher rates of MASH resolution without worsening of fibrosis (44%, 56% and 62%, respectively) than placebo (10%). Improvement in fibrosis by at least one stage was observed in 55% of participants in the 5 mg group, 51% in the 10 mg group, 51% in the 15 mg group and 30% in the placebo group. However, the statistical significance of these improvements in liver fibrosis has not been formally tested after adjustment for multiple comparisons. In addition, tirzepatide significantly reduced body weight and plasma lipid and HbA_1c_ levels compared with placebo [43]. These findings warrant further investigation in Phase III studies.

### GLP-1/glucagon receptor co-agonists

Co-administration of GLP-1 and glucagon is more effective than GLP-1 alone at reducing food intake, increasing energy expenditure and hepatic fatty acid oxidation and decreasing hepatic lipid content [44]. Several GLP-1/glucagon receptor co-agonists are in clinical development for the treatment of MASH with moderate-to-advanced fibrosis, including cotadutide, pemvidutide and survodutide. For example, in a Phase IIb trial involving 293 adults with obesity and biopsy-confirmed MASH and fibrosis stage F1–F3, compared with placebo (14%), once-weekly subcutaneous survodutide at doses of 2.4 mg, 4.8 mg, or 6.0 mg for 24 weeks resulted in significantly greater improvements in MASH with no worsening of fibrosis (47%, 62% and 43%, respectively). Improvement in liver fibrosis was observed in 34% of the 6.0 mg group compared with 22% in the placebo group. Survodutide significantly reduced body weight and plasma lipid and HbA_1c_ levels compared with placebo [45]. These findings warrant further investigation in Phase III studies.

### Fibroblast growth factor 21 analogues

Fibroblast growth factor 21 (FGF21) is a hepatokine with multiple beneficial effects on metabolism [46, 47]. FGF21 analogues increase energy expenditure, improve insulin resistance and dyslipidaemia and increase serum adiponectin levels. In a Phase IIb trial involving 219 participants with obesity and biopsy-confirmed MASH and stage F2 or F3 fibrosis, subcutaneous pegozafermin at 15 mg or 30 mg weekly or 44 mg every 2 weeks for 24 weeks resulted in higher rates of MASH resolution (37%, 23% and 25%, respectively) than placebo (2%). Liver fibrosis improved in 25% and 44% of participants in the 30 mg and 44 mg pegozafermin groups, respectively, compared with 7% in the placebo group [48]. In a Phase IIb trial involving 128 adults with obesity and biopsy-confirmed MASH and stage F2 or F3 fibrosis, subcutaneous efruxifermin at 28 mg or 50 mg weekly for 96 weeks resulted in higher rates of improvement in fibrosis of one or more stage without worsening of MASH (46% and 75%, respectively) than placebo (24%) [49]. In both trials, changes in body weight and HbA_1c_ levels did not differ significantly between the FGF21 and placebo treatment groups. These findings require further investigation in Phase III trials.

### Resmetirom

Activation of THR-β in the liver improves mitochondrial function, thereby lowering hepatic lipid content and reducing lipotoxicity [50]. In the Phase III placebo-controlled MAESTRO-NASH trial involving 966 participants with obesity and biopsy-confirmed MASH and fibrosis stage F1B, F2 or F3, 80 mg and 100 mg of resmetirom once daily for 52 weeks resulted in significantly higher rates of MASH resolution without worsening of fibrosis (26% and 30%, respectively) than placebo (10%). Improvement in fibrosis by one or more stage without worsening of MASH was also significantly greater in the 80 mg (24%) and 100 mg (26%) resmetirom groups than in the placebo group (14%). Resmetirom improved plasma concentrations of LDL-cholesterol, triacylglycerol and lipoprotein(a) but had neutral effects on body weight, HbA_1c_ and insulin resistance and did not induce adverse endocrine events, tachyarrhythmias or major changes in bone mineral density [51]. Based on these results, resmetirom was conditionally approved by the FDA on 14 March 2024 [52] and by the EMA on 19 August 2025 [53] for the treatment of adults with non-cirrhotic MASH and moderate-to-advanced fibrosis.

### Pioglitazone

Peroxisome proliferator-activated receptors (PPARs) are a group of nuclear receptors that play important roles in modulating glucose and lipid metabolism. They also regulate inflammatory and fibrotic processes. Three PPAR isotypes exist (α, β/δ and γ) [54]. The PPAR-γ agonist pioglitazone is approved for the treatment of type 2 diabetes [54]. It is also protective against acute myocardial infarction and ischaemic stroke [55]. A meta-analysis of five Phase IIb randomised trials (315 participants with and without type 2 diabetes) found that pioglitazone treatment (up to 45 mg/day) for 6–24 months significantly improved liver histology in participants with biopsy-confirmed MASH and liver fibrosis [56]. ORs of 3.65 (95% CI 2.32, 5.74) for MASH resolution and 4.53 (95% CI 1.52, 13.52) for improvement in advanced fibrosis (stage F3 or F4) were reported compared with control groups [56]. Moderate weight gain (up to nearly 2.5% from baseline), especially in subcutaneous fat rather than visceral fat, which is often observed during pioglitazone treatment [57], was also reported in this meta-analysis [56].

### Lanifibranor

The pan-PPAR (PPARα, PPARδ and PPARγ) agonist lanifibranor (800 mg or 1200 mg daily for 24 weeks) was evaluated in a Phase IIb placebo-controlled clinical trial in 247 participants with obesity and biopsy-proven MASH and liver fibrosis. A significant decrease of at least 2 points in the histological SAF-A score (the activity component of the Steatosis, Activity, Fibrosis [SAF] scoring system that incorporates scores for ballooning and inflammation) without worsening of liver fibrosis was observed in 55% of participants treated with 1200 mg of lanifibranor compared with 33% in the placebo group. Improvement in liver fibrosis without worsening of MASH was observed in 48% of participants in the lanifibranor 1200 mg group compared with 29% in the placebo group. Lanifibranor improved plasma lipids, insulin resistance and HbA_1c_ levels and moderately increased body weight (up to nearly 2.5%) compared with placebo [58]. These findings warrant further investigation in Phase III studies.

## Precision medicine for MASLD/MASH treatment

Although the pharmacological treatments discussed in the previous sections have shown promising results, the majority of individuals with MASLD/MASH do not benefit from these treatments as expected in terms of improving the liver phenotype. The potential reasons for this, which are relevant to the future implementation of precision medicine in MASLD, are discussed below.

### Barriers to pharmacological treatment of MASLD

Some medications are discontinued early because of side effects or undergo inadequate dose escalation. This applies particularly to GLP-1 receptor agonist-based therapies, which are highly effective in the treatment of obesity and MASLD/MASH [59] but are associated with gastrointestinal side effects that lead to premature discontinuation or failure to increase the weekly dose as recommended. For example, in a network meta-analysis that included 33,354 participants, nausea, vomiting, diarrhoea and constipation were the most common gastrointestinal side effects associated with GLP-1 receptor agonists, with a nearly two- to fivefold increased risk compared with control [60]. Other, mostly very rare, side effects of GLP-1 receptor agonist-based therapies, such as non-arteritic anterior ischaemic optic neuropathy, deserve further investigation [61]. Regarding SGLT2 inhibitors, in the Phase III DEAN trial in participants with MASH, the safety profile of dapagliflozin was favourable and consistent with that observed in previous studies. Adverse events were less frequent with dapagliflozin than placebo (56% vs 64%), and no serious adverse events were reported in the dapagliflozin group [32]. In RCTs of FGF21 analogues in participants with MASH, the most common adverse events reported were nausea, diarrhoea and injection site erythema [48, 49]. For resmetirom, the most frequent adverse events reported are diarrhoea and nausea. The resmetirom-induced decrease in serum free T4 levels and increase in circulating sex hormone-binding globulin levels [51] warrant further investigation. For pioglitazone, in addition to moderate weight gain, other side effects reported include bone loss and a risk of bladder cancer, which is believed to be low (i.e. one additional case of bladder cancer for every 899–6380 individuals treated for 3 or more years). Furthermore, fluid retention-associated heart failure may also occur in individuals with pre-existing systolic or diastolic dysfunction, although pioglitazone has been found to improve left ventricular function [57].

Although these metabolism-based therapies produce rapid beneficial effects on glucose and lipid metabolism and body weight, a change in liver phenotype requires longer treatment periods. These divergent effects may be particularly relevant for GLP-1 receptor agonist-based therapies, SGLT2 inhibitors and FGF21 analogues, and could lead to premature discontinuation of drug therapy because of suspected low efficacy. The longer treatment periods required for changes in liver phenotype also suggest the need for longer study periods in future clinical trials. Furthermore, to date, the two drugs conditionally approved for the treatment of MASH (resmetirom and semaglutide 2.4 mg/week), and other drugs in the process of being approved, are expensive, and healthcare providers may be reluctant to initiate treatment or continue treatment for longer periods. Finally, genetically based biological non-response to pharmacological treatment may also occur, for example as seen with GLP-1 receptor agonist-based therapies [62].

### Heterogeneity in the pathogenesis and cardiometabolic risk of MASLD

In most cases, excessive intake of glucose and fructose promotes fatty acid production through hepatic de novo lipogenesis. Together with a higher intake of saturated fatty acids, this process leads to low-grade inflammation in adipose tissue and the liver, as well as insulin resistance in adipose tissue, the liver and skeletal muscle. However, recognising the substantial heterogeneity underlying MASLD pathophysiology is crucial for improving risk stratification and informing therapeutic strategies. Numerous frameworks have been proposed for the classification of individuals with MASLD based on clinical characteristics, laboratory parameters and genetic factors. Three of the most well-established contributors to MASLD pathogenesis have been identified as MASLD with a dominant hepatic genetic component, MASLD with a metabolic component related to hepatic de novo lipogenesis, and MASLD with a metabolic component related to adipose tissue dysfunction [22]. However, a key challenge in stratifying people into distinct subgroups is the substantial overlap among dietary, anthropometric, clinical and genetic factors, which rarely operate in isolation. Consequently, most individuals exhibit contributions from multiple pathogenic drivers. Despite this complexity, identifying dominant disease mechanisms in individual patients may support the development and implementation of more precise, mechanism-based therapeutic strategies for MASLD.

There is substantial heterogeneity not only in the pathogenesis of MASLD but also in its long-term clinical outcomes. Recent studies have identified distinct MASLD subtypes associated with varying hepatic and, more importantly, cardiometabolic risks [63–65]. These findings support the idea that, in the future, data dimensionality reduction approaches based on clustering strategies might improve the prediction of MASLD-associated risk; such approaches are already being used in obesity and type 2 diabetes [66]. This understanding of heterogeneity in the pathogenesis and cardiometabolic risk of MASLD may also lead to personalised treatments. However, in some individuals, the pathogenesis of MASLD remains unclear. In this regard, combination therapies may be considered. First results from Phase I and Phase IIa clinical trials indicate that combination therapies are more effective than single pharmacological approaches in improving clinical parameters of MASLD [67, 68]. Currently, semaglutide and resmetirom are the only two FDA and EMA conditionally approved pharmacotherapies for non-cirrhotic MASH and liver fibrosis that target complementary aspects of the disease. Resmetirom acts on hepatocytes via the THR-β receptor to reduce hepatic fat content, improve mitochondrial function and alleviate liver inflammation and fibrosis, whereas semaglutide acts systemically, outside the liver, to reduce energy intake, promote weight loss and improve insulin sensitivity [68, 69]. Therefore, this combination therapy, often described as pairing a liver-directed therapy (resmetirom) with a systemic metabolic agent (semaglutide), represents a promising, potentially more effective or optimal therapeutic approach than monotherapy in individuals with/without type 2 diabetes who have coexisting MASH [67–69]. To date, however, no clinical trials have compared combined therapy with semaglutide 2.4 mg/week and resmetirom with the individual therapies for the treatment of MASH and moderate-to-advanced fibrosis.

## Conclusions

MASLD is highly prevalent among individuals with type 2 diabetes worldwide and is associated with liver-related complications, hepatocellular carcinoma, cardiovascular disease and certain extrahepatic cancers (mainly non-liver gastrointestinal cancers). First-line treatment for MASLD includes a weight-reducing diet, increased physical activity levels, alcohol avoidance, and effective management of obesity, type 2 diabetes and other metabolic comorbidities. To date, subcutaneous semaglutide 2.4 mg/week and resmetirom are the only two medications conditionally approved by the FDA and EMA for the treatment of adults with non-cirrhotic MASH and moderate-to-advanced fibrosis. Phase III randomised, placebo-controlled trials of dual or triple incretin receptor co-agonists, pan-PPAR agonists (lanifibranor) and FGF21 analogues are ongoing. For individuals with type 2 diabetes and MASLD/MASH, treatment should preferably include GLP-1 receptor agonist-based therapies and SGLT2 inhibitors, which have been shown to improve MASLD/MASH and provide established cardiorenal benefits.

## Supplementary Information

Below is the link to the electronic supplementary material.

Slideset of figures (PPTX 503 KB)

## Abbreviations

ELF: Enhanced liver fibrosis
EMA: European Medicines Agency
FDA: Food and Drug Administration
FGF21: Fibroblast growth factor 21
FIB-4: Fibrosis 4
FXR: Farnesoid X receptor
GIP: Glucose-dependent insulinotropic polypeptide
GLP-1: Glucagon-like peptide-1
MASH: Metabolic dysfunction-associated steatohepatitis
MASLD: Metabolic dysfunction-associated steatotic liver disease
MRE: Magnetic resonance elastography
NAFLD: Non-alcoholic fatty liver disease
PPAR: Peroxisome proliferator-activated receptor
SAF score: Steatosis, activity, and fibrosis score
SGLT2: Sodium–glucose cotransporter 2
THR: Thyroid hormone receptor
VCTE: Vibration-controlled transient elastography
